# Identification of a t(3;4)(p1.3;q1.5) translocation breakpoint in pigs using somatic cell hybrid mapping and high-resolution mate-pair sequencing

**DOI:** 10.1371/journal.pone.0187617

**Published:** 2017-11-09

**Authors:** Katia Fève, Sylvain Foissac, Alain Pinton, Florence Mompart, Diane Esquerré, Thomas Faraut, Martine Yerle, Juliette Riquet

**Affiliations:** GenPhySE, Université de Toulouse, INRA, INPT, ENVT, Castanet-Tolosan, France; University of Helsinki, FINLAND

## Abstract

Reciprocal translocations are the most frequently occurring constitutional structural rearrangements in mammalian genomes. In phenotypically normal pigs, an incidence of 1/200 is estimated for such rearrangements. Even if constitutional translocations do not necessarily induce defects and diseases, they are responsible for significant economic losses in domestic animals due to reproduction failures. Over the last 30 years, advances in molecular and cytogenetic technologies have led to major improvements in the resolution of the characterization of translocation events. Characterization of translocation breakpoints helps to decipher the mechanisms that lead to such rearrangements and the functions of the genes that are involved in the translocation. Here, we describe the fine characterization of a reciprocal translocation t(3;4) (p1.3;q1.5) detected in a pig line. The breakpoint was identified at the base-pair level using a positional cloning and chromosome walking strategy in somatic cell hybrids that were generated from an animal that carries this translocation. We show that this translocation occurs within the *ADAMTSL4* gene and results in a loss of expression in homozygous carriers. In addition, by taking this translocation as a model, we used a whole-genome next-generation mate-pair sequencing approach on pooled individuals to evaluate this strategy for high-throughput screening of structural rearrangements.

## Introduction

Reciprocal translocations are the most frequently occurring constitutional structural rearrangements both in humans, with an incidence ranging from 1/752 live-births [1] to 1/250 for prenatal diagnosis [2], and pigs with an incidence of 1/200 in phenotypically normal pigs [3]. As in humans, constitutional reciprocal translocations have no phenotypic effect unless the breakpoint is located within a developmentally important gene or regulatory sequence. Nevertheless, they can give rise to reproductive failure by interfering with the proper segregation of chromosomes during meiosis [4]. Because of their potential impact on reproduction, reciprocal translocations can be responsible for significant economic losses in domestic animal breeding [5,6]. In pigs and cattle, due to the increased use of artificial insemination (AI) since the end of the 1980’s, screening campaigns to detect and eradicate chromosomal abnormalities have been more largely implemented in these species [3]. To date, more than 164 translocations are described in pigs, and all chromosomes and breeds are affected by this phenomenon. In of spite their apparent prevalence and functional importance, the mechanism of the formation of reciprocal translocations and their frequencies in different populations as well as their relative impact on fertility or on other traits remain poorly understood [7,8].

The first chromosome rearrangement involving large DNA fragments in pigs was identified and described by [8] using conventional chromosome staining techniques. The application of banding techniques developed in humans [9,10] to domestic animal species [11], and later of fluorescent in situ hybridization techniques (reviewed in [12]), have allowed the detection of numerous new translocations [13,14]. However, these conventional cytogenetic approaches performed on metaphase chromosomal spreads are limited to the genome-wide detection of chromosomal abnormalities with a resolution of 5 to 10 Mb. In humans, the instrumental value of disease-associated balanced chromosomal rearrangements (DBCR) for positional cloning of disease-related genes spurred high-resolution characterization of DBCR breakpoints [15], starting with FISH using Bacterial Artificial Chromosome (BAC) as probes, followed by array painting [16] and, more recently, next-generation sequencing techniques that can locate the breakpoint to the base-pair level [17]. The precise delineation of translocation breakpoints provides molecular information, and potentially identifies molecular defects, which can contribute to understanding the mechanisms that underlie such genomic events [7]. Furthermore, it enables the development of molecular-based diagnostic tests for population screening.

Here, we report the characterization of a reciprocal translocation t(3;4) (p1.3;q1.5) that segregates in a Large-White pig line, which has been selected since six generations for residual feed intake (RFI) [18]. The translocation breakpoints for both chromosomes were identified at the base-pair level using a positional cloning strategy in somatic cell hybrids that were generated from a t(3;4) (p1.3;q1.5) carrier and their location was further confirmed by using data from whole-genome next-generation mate-pair sequencing. This translocation is located within the *ADAMTSL4* gene and thus modifies its expression in translocation carriers. The flanking sequences were used to develop a PCR-based diagnostic test to genotype all the animals of the selected line.

## Materials and methods

### Ethics statement

Our experiments were conducted in accordance with the European Directive 2010/63/EU on the protection of animals used for scientific purposes, and approved by the Ethics Committee for Animal Experimentation of the Poitou Charentes region (France) (N°CE2012-2). The animals used in this study were raised in a conventional production system with little or no animal manipulation. The animals are checked for health and well-being every day, and they are prided with food and water ad libitum. Animals were sacrificed in a commercial slaughterhouse following national and institutional guidelines for the Good Experimental Practices and approved by the Ethical Committee of INRA (French National Institute for Agricultural Research). The experimentation registration number for the experimental farm on which the animals were raised is A-17-661.

### Animals and cytogenetic analysis

**Fig 1:**
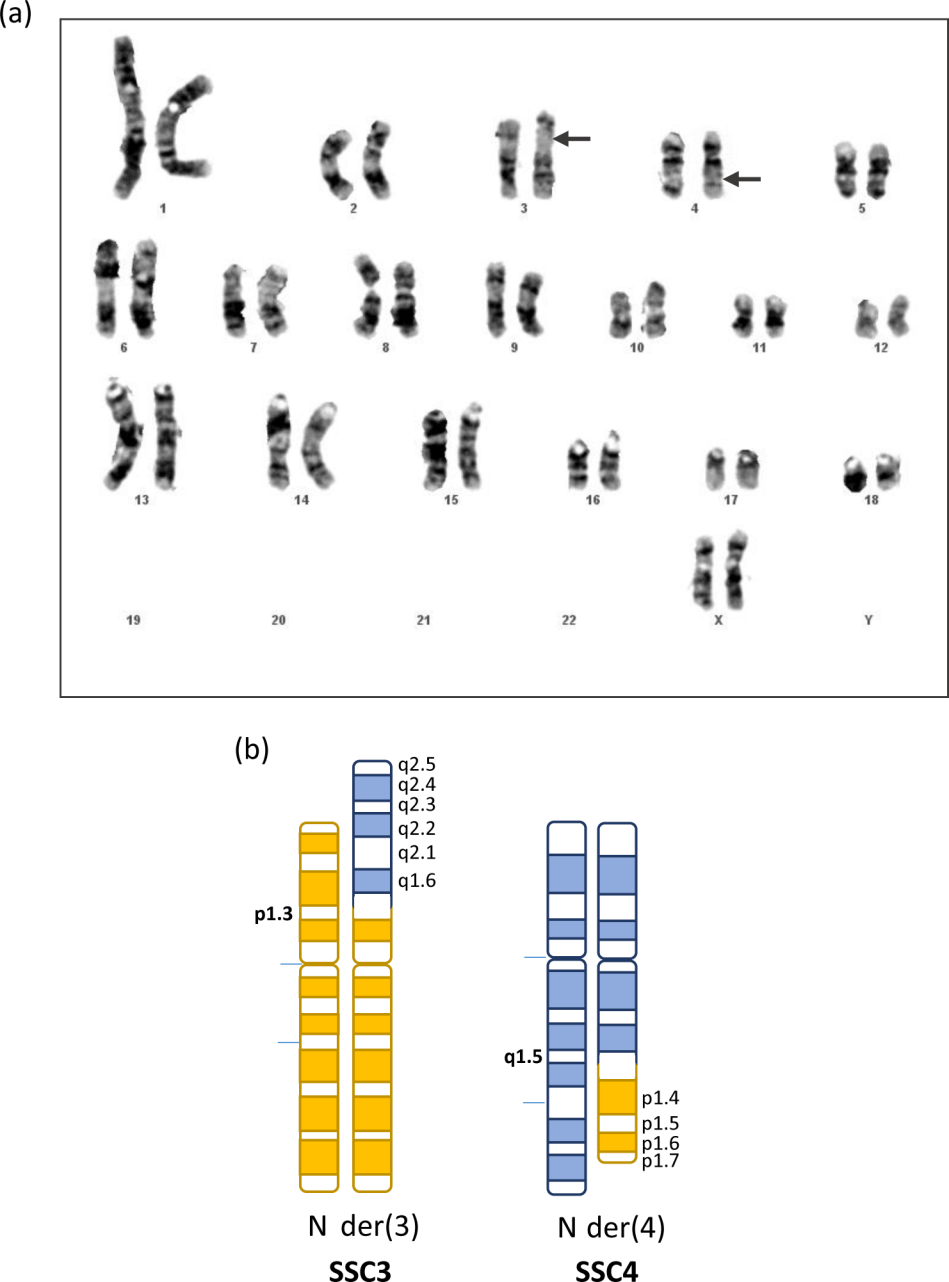
Karyotype of the balanced reciprocal translocation t(3;4)(p1.3;q1.5). (a): G-band karyotype obtained from a LW female, heterozygous for the translocation. The breakpoint positions on SSC3 and SSC4 are indicated with arrows. (b): Schematic representation of SSC3 and SSC4 pairs including one normal SSC3 or SSC4 chromosome (N) and one translocated chromosome der(3) or der(4).

The reciprocal translocation was identified in a Large-White pig line produced on an INRA experimental facility (Rouille-GenESI, Vienne, France). Metaphase spreads were obtained from classic lymphocyte cultures and subjected to GTG-banding as previously described [13]. The chromosomal rearrangement was described, according to the standard nomenclature, as 38,XY,t(3;4)(p1.3;q1.5) (Fig 1). Chromosomal analyses were performed for 111 animals from this line, which were candidates to generate the next generation.

### Production of somatic cell hybrids and cytogenetic characterization

#### Hybrid clone production

Somatic cell hybrid clones were produced by fusing 20.10^6^ lymphocytes of a homozygous t(3;4) (p1.3;q1.5) translocation carrier with 20.10^6^ recipient hamster cells from the permanent HPRT- Chinese hamster line Wg3hCl2. Fusion and selection were based on standard methods [19]. The selected hybrid clones were grown in RPMI 1640 medium containing hypoxanthine, aminopterin and thymidine (HAT). Genomic DNA of the hybrid cell lines was isolated using the DNeasy Blood & Tissue Kit (QIAGEN).

#### Production of pIRS-PCR (or porcine interSINE) probes and FISH experiments

Probes for FISH experiments were generated using 50 ng of hybrid DNA as template for interSINE-PCR amplification as described previously [20]. For each probe, 2 μl of pIRS-PCR amplified hybrid DNA were labeled by random priming with biotin-16-dUTP using the BioPrime labeling system (Invitrogen). The probes were precipitated in the presence of competitor DNA (5 μg of porcine Cot-1 DNA). The DNA pellets were resuspended in 25 μl of hybridization mixture, denaturated and preannealed for 3 h at 37°C to block repetitive sequences. FISH experiments were performed according to [21]. The probes were detected using Alexa 594 conjugated to Streptavidin (Molecular Probes, Eugene, OR, USA) [20].

Painted chromosomes or fragments of chromosomes were identified by comparison with G-banded metaphase chromosome pictures taken before hybridization [20].

### Chromosome walking and STS selection

Microsatellite markers were chosen on the genetic maps of *Sus scrofa* (SSC) chromosomes SSC3 and SSC4. In a second step, the SNP sequences of the Illumina pig 60K SNP chip were used as STSs and pairs of primers were defined based on the sequences on either side of each SNP (S1 Table). In a third step, new SSC3 and SSC4 STSs were defined in the selected intervals using the pig Sscrofa 10.2 draft sequence as reference (http://www.ensembl.org/Sus_scrofa/Info/Index). Primers were selected using Primer3 software, and PCR conditions were determined for each STS (S1 Table).

### Sequencing of the t(3;4) breakpoint

Junction fragments across the t(3;4) breakpoint were amplified by long-range PCR using the *GeneAmp*® XL PCR Kit (Life Technologies). Products from these reactions were purified by incubation with 10 units of Exonuclease I and 0.5 unit of SAP at 37 C during 45 min followed by heat inactivation (80 C during 30 min). They were then sequenced using the ABI PRISM® BigDye® Terminator v3.1 Cycle Sequencing Kit on an ABI PRISM 3730 sequencer (Applied Biosystems, Foster City, CA, USA). Computational analysis of the resulting sequences allowed precise localization of the breakpoints on SSC3 and 4.

### PCR validations

#### Genotyping

Two pairs of primers were selected respectively on SSC3 (*Trsl_SSC3*.*Up and Trsl_SSC3*.*Dn*) and SSC4 (*Trsl_SSC4*.*Up and Trsl_SSC4*.*Dn*) to amplify the junction fragments across the t(3;4) breakpoint. In order to genotype animals for the translocation (homozygous or heterozygous), only three primers were used: the unique forward primer *Trsl_SSC4*.*Up* was used with *Trsl_SSC4*.*Dn* (amplification of a 346-bp fragment in the absence of translocation) or with *Trsl_SSC3*.*Dn* (amplification of a fragment of 277 bp in the presence of the translocation) (S1 Fig and S2A Fig). Genotyping was performed with 10 ng of DNA in the presence of 0.1 μM of each primer, 1.5 mM of MgCl_2_ for 35 cycles at 58 C.

#### Expression analysis

Among the animals of the LW line, three animals, each one with one of the three possible translocation genotypes were selected: lung and heart tissue samples were collected when animals were slaughtered. Total RNA was extracted for each tissue using the guanidium thiocyanate method [22] and treated with DNAse. The quality of the RNA was analyzed by gel electrophoresis and for each sample, 2 μg of total RNA was reverse-transcribed using a poly-T oligonucleotide, according to the manufacturer’s instructions. Two pairs of primers were defined in the porcine sequence according to the human annotation of the gene: ADAM_cDNA in exons 13 (Up: GTCTCCAACCGGATACTGGA) and 14 (Dn: TACCAGGGCCACAGGAAC), and ADAM_cDNA_control in exons 15 (Up: TGATGAAGTGAGCGAGCAAG) and 17 (Dn: CCAGTTTGGACACACAGATGA) (S3 Fig). PCR amplifications were performed in standard conditions for 38 cycles at 58 C in the presence of 1.5 mM of MgCl_2_.

### Mate-paired sequencing

The pooled genomic DNA of five Large White animals (50 ng in total), one of which was heterozygous for the t(3;4)(p1.3;q1.5) translocation, was used to generate a mate-pair library following the Nextera protocol from Illumina. The target insert size of 6 kb was selected and the library was sequenced with read lengths of 100 bp for each mate. A second pool of five normal Duroc animals, which are assumed to harbor only normal chromosomes with respect to the translocation, was used as a reference normal sample. Sequencing data of the Duroc and Large-White pooled samples are publicly available in the Sequence Read Archive (SRA) database under accession numbers SRX2996560 and SRX2996558, respectively. Sequences were trimmed for adapters using cutadapt [23]. The reverse complement sequences were aligned to the Sscrofa10.2 genome assembly using bwa [24] and alignments with a mapping quality above 10 were kept for further analysis. We used three different software to identify structural variants (SV) from this mate-pair sequencing data: delly (doi: 10.1093/bioinformatics/bts378), SVDetect [25] and GASVPro [26]. All three software were used with default parameters. Only translocations that were supported by more than 10 mate-pairs were considered as reliable candidate translocations. For SVDetect, the set of candidate translocations was limited to balanced translocations.

## Results

### Production and characterization of somatic cell hybrids

The balanced t(3;4) (p1.3;q1.5) was originally detected by G-banded karyotype analysis in a LW pig with a small litter size (Fig 1). The complete karyotypes of 110 additional animals, which are reproductive candidates for the next generation, were established and the segregation of the balanced translocation in this population was confirmed. Among the 111 animals tested, 29 carried the chromosomal rearrangement (27 heterozygotes and 2 homozygotes). To localize the breakpoint junctions on SSC3 and 4, interspecific somatic cell hybrid clones were produced. Porcine lymphocyte cells obtained from one of the two homozygous females were fused to a permanent Chinese hamster cell line. In total, 12 independent hybrid clones were produced and genomic DNA from each clone was prepared for further characterization.

To verify the presence of porcine DNA in the hybrids and select the clones containing the translocated chromosomes, a set of 37 microsatellites was defined and used for PCR experiments on the DNA of each clone. The set included 12 microsatellites that are located on 12 different chromosomes on the porcine genetic map [27], plus 12 and 13 microsatellites located respectively on SSC3 and SSC4 genetic maps (Table 1). As a first step, the presence of porcine chromosomal material in the hybrid clones was confirmed using the 12 microsatellites localized on different chromosomes. Amplification products for one to four markers were obtained for each clone. The presence of SSC3 and SSC4 material was then tested using SSC3 and SSC4 specific microsatellites. No amplification was obtained for four hybrids. Among the remaining eight hybrid clones, seven (Hb1.4; Hb1.5; Hb1.7; Hb1.8; Hb1.9; Hb2.2; Hb2.3) were positive for 13 microsatellites of the 25 specific SSC3/SSC4 microsatellites tested and one hybrid clone (Hb2.5) was positive for all microsatellites. A summary of these results is in Table 1 and shows that four clones did not contain any of the two translocated chromosomes, seven clones were identical and contained the same translocated derivative chromosome der(4) and, in contrast, the second rearranged chromosome der(3) was never retained alone and was only present in clone Hb2.5 which also contained the der(4) chimeric copy.

**Table 1:** Results obtained by PCR-screening of each hybrid clone with microsatellite markers.

	*Chr*	*Genetic position (cM)*	*Physical position on Sscrofa10.2 (Mb)*	*Hb* *1.8*	*Hb* *2.5*	*Hb* *1.3*	*Hb* *1.4*	*Hb* *1.5*	*Hb* *1.6*	*Hb* *1.7*	*Hb* *1.9*	*Hb* *2.1*	*Hb* *2.2*	*Hb* *2.3*	*Hb* *2.4*
*S0142*	1	83	-	1	1	0	0	0	0	0	0	0	0	0	0
*SWR2516*	2	1	-	0	0	0	0	0	0	1	0	1	1	0	1
*SW492*	6	69	-	0	0	1	0	0	1	0	0	0	0	1	0
*SW1856*	7	64	-	0	0	0	0	1	0	0	1	0	0	1	0
*SWC19*	10	50	-	0	0	0	0	1	1	0	0	1	0	1	1
*SW168*	12	70	-	0	0	1	0	0	0	0	0	0	0	0	0
*S0084*	13	61	-	0	0	0	0	0	0	0	0	0	0	0	0
*SW1125*	14	22	-	0	0	0	0	0	0	0	0	0	0	0	0
*S0004*	15	16	-	0	0	1	0	0	1	0	0	0	0	0	0
*SW1341*	16	40	-	1	0	1	1	1	1	1	0	1	0	1	1
*SWR1004*	17	15	-	0	0	0	0	0	0	0	0	0	0	0	0
*S0062*	18	43	-	0	0	0	0	0	0	0	0	0	0	0	0
*S0213*	3	7.8	-	1	1	0	1	1	0	1	1	0	1	1	0
*Sw2429*	3	17	-	1	1	0	1	1	0	1	1	0	1	1	0
*SW251*	3	42,3	21,948	1	1	0	1	1	0	1	1	0	1	1	0
*SW2527*	3	42.3	?	1	1	0	1	1	0	1	1	0	1	1	0
*SW487*	3	42.8	31,261	0	1	0	0	0	0	0	0	0	0	0	0
*S0174*	3	43	-	0	1	0	0	0	0	0	0	0	0	0	0
*SW1525*	3	45	-	0	1	0	0	0	0	0	0	0	0	0	0
*SW860*	3	47	-	0	1	0	0	0	0	0	0	0	0	0	0
*S0034*	3	49.8	-	0	1	0	0	0	0	0	0	0	0	0	0
*S0032*	3	50.6	-	0	1	0	0	0	0	0	0	0	0	0	0
*SW1432*	3	51	-	0	1	0	0	0	0	0	0	0	0	0	0
*SW2597*	3	52	-	0	1	0	0	0	0	0	0	0	0	0	0
*SW45*	4	55.9	-	1	1	0	1	1	0	1	1	0	1	1	0
*S0217*	4	69.6	-	1	1	0	1	1	0	1	1	0	1	1	0
*S0073*	4	74.4	-	1	1	0	1	1	0	1	1	0	1	1	0
*S0764*	4	75	-	1	1	0	1	1	0	1	1	0	1	1	0
*SW1996*	4	77	-	1	1	0	1	1	0	1	1	0	1	1	0
*SW286*	4	78.3	-	1	1	0	1	1	0	1	1	0	1	1	0
*Sw270*	4	79.3	-	1	1	0	1	1	0	1	1	0	1	1	0
*S0214*	4	79.3	-	1	1	0	1	1	0	1	1	0	1	1	0
*SW512*	4	80.5	106,511	1	1	0	1	1	0	1	1	0	1	1	0
*SW524*	4	99.3	?	0	1	0	0	0	0	0	0	0	0	0	0
*SW2435*	4	102.8	120,509	0	1	0	0	0	0	0	0	0	0	0	0
*S0067*	4	102.8	-	0	1	0	0	0	0	0	0	0	0	0	0
*SW2066*	4	121	-	0	1	0	0	0	0	0	0	0	0	0	0

List of the microsatellites used to characterize the pig content in each hybrid clone. For each marker, its location on the genetic map and the amplification results obtained (0: absence, 1: presence) are reported. For the most proximal microsatellites on both sides of the translocation point, positions on the V10.2 version of the pig draft sequence are indicated.

**Fig 2:**
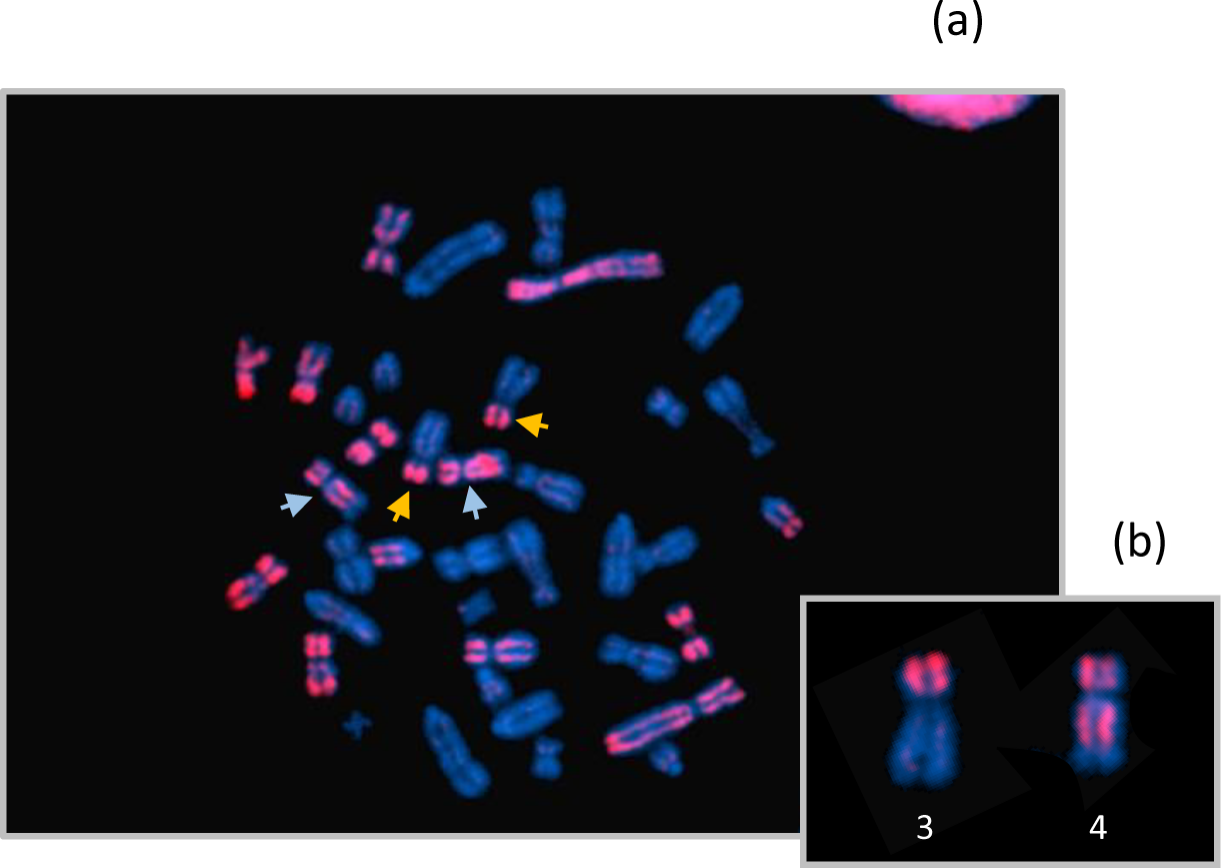
Fluorescent in situ hybridization of the pIRS-PCR probe, derived from hybrid Hb1.8, on normal pig metaphase chromosomes. pIRS-PCR products label the entire length of chromosomes 1, 8, 10, 16, X, and partially chromosomes 3, 4 and 5. Chromosomes 3 and 4 are indicated with yellow and blue arrows respectively (a), and are magnified in (b). On these two chromosomes, signals are observed on the p arm for SSC3 and in the p15-q22 region for SSC4, confirming the presence of the translocated derivative chromosome der(4) in Hb1.8 hybrid clone.

To confirm these PCR results, pIRS-PCR (porcine Interspersed Repetitive Sequence) products were prepared using the DNA of Hb1.8 (chosen among the seven clones carrying the der(4) chromosome) and Hb2.5 clones as probes in FISH experiments on porcine metaphase chromosomes as described previously [20]. pIRS-PCR revealed all the porcine chromosome fragments that were retained in addition to the hamster genome in these clones. More precisely, a pIRS-PCR probe prepared from Hb1.8 revealed a clear signal on five whole porcine chromosomes (SSC1, SSC8, SSC10, SSC16 and SSCX), on SSC5 p15-q23 and finally on the p-arm of SSC3, and on SSC4 p15-q22, (Fig 2A and 2B). Similarly, porcine metaphase spreads hybridized with the Hb2.5 pIRS-PCR probe revealed a clear signal on SSC3 and SSC4 chromosomes (data not shown). These FISH experiments confirmed the results obtained by PCR screening with the 25 microsatellites. Among the different hybrid clones generated, only Hb1.8 and Hb2.5 were retained for the subsequent steps of the characterization of the breakpoints.

### Localization of breakpoints by mapping STS markers on Hb1.8 and Hb2.5 hybrid clones

The first localizations of breakpoints were obtained by using the 25 microsatellites initially used to characterize the presence of SSC3 and SSC4 in the somatic hybrid clones (Table 1). The four microsatellite markers localized in the region between 0 and 42 cM on the SSC3 genetic map were amplified using Hb1.8 DNA, whereas with the remaining eight markers selected for SSC3 there was no amplification. The same PCR tests were carried out on Hb2.5 hybrid clone DNA as a positive control since it contains the two rearranged chromosomes. Based on the SW2527 and SW487 microsatellites that map to positions 42.3 cM and 42.8 cM respectively on SSC3, a first sub-1-cM interval containing the breakpoint was defined on this chromosome. All the SSC4 markers located between 55.9 cM and 80.5 cM were positive using Hb1.8 hybrid DNA as the PCR template. The first marker (SW524), which gave no amplification product, was localized at 99.3 cM and led to the identification of a minimum interval of 19 cM on SSC4. To define the position of these intervals on the porcine draft sequence (Sscrofa10.2), the four microsatellite markers that are at each end of the two intervals were searched in the available draft sequence. Two of the four microsatellites were mapped on the sequence: SW487 at 31.261 Mb on SSC3 and SW512 at 106.511 Mb on SSC4 (Table 1).

**Fig 3:**
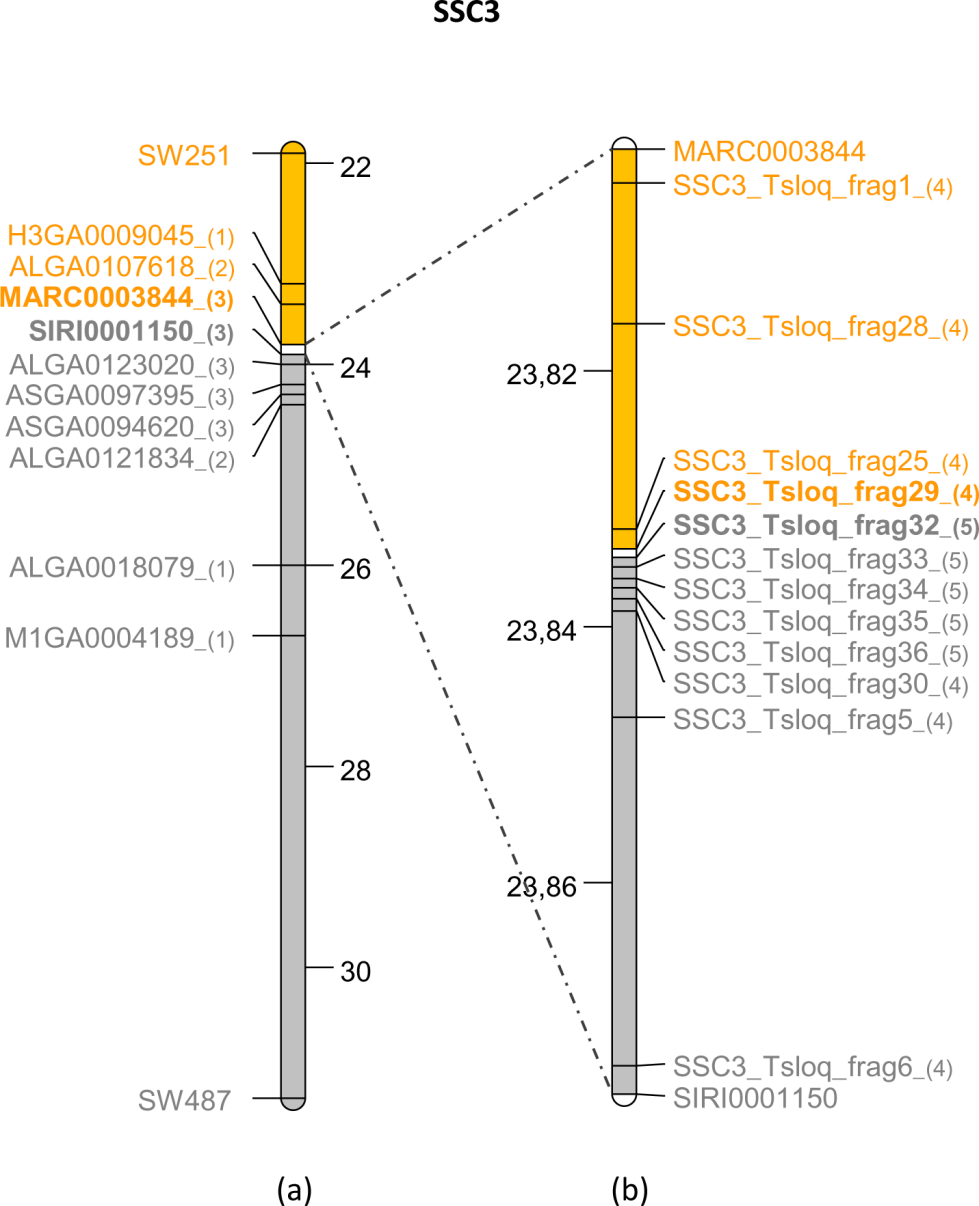
Map of the SSC3 region containing the breakpoint. All the markers (microsatellites, SNPs and STS) used in each round of PCR amplification are indicated on the map corresponding to the 22–32 Mb interval (a) or on a zoom of the 23.80–23.87 Mb interval (b). The successive batches of STS are indicated by a number in brackets following the STS names. Screening results obtained for each marker on Hb1.8 are represented by colors (yellow: positive, grey: negative). The smallest interval on the SSC3 map is highlighted in white and the names of the two adjacent STS are indicated in bold.

**Fig 4:**
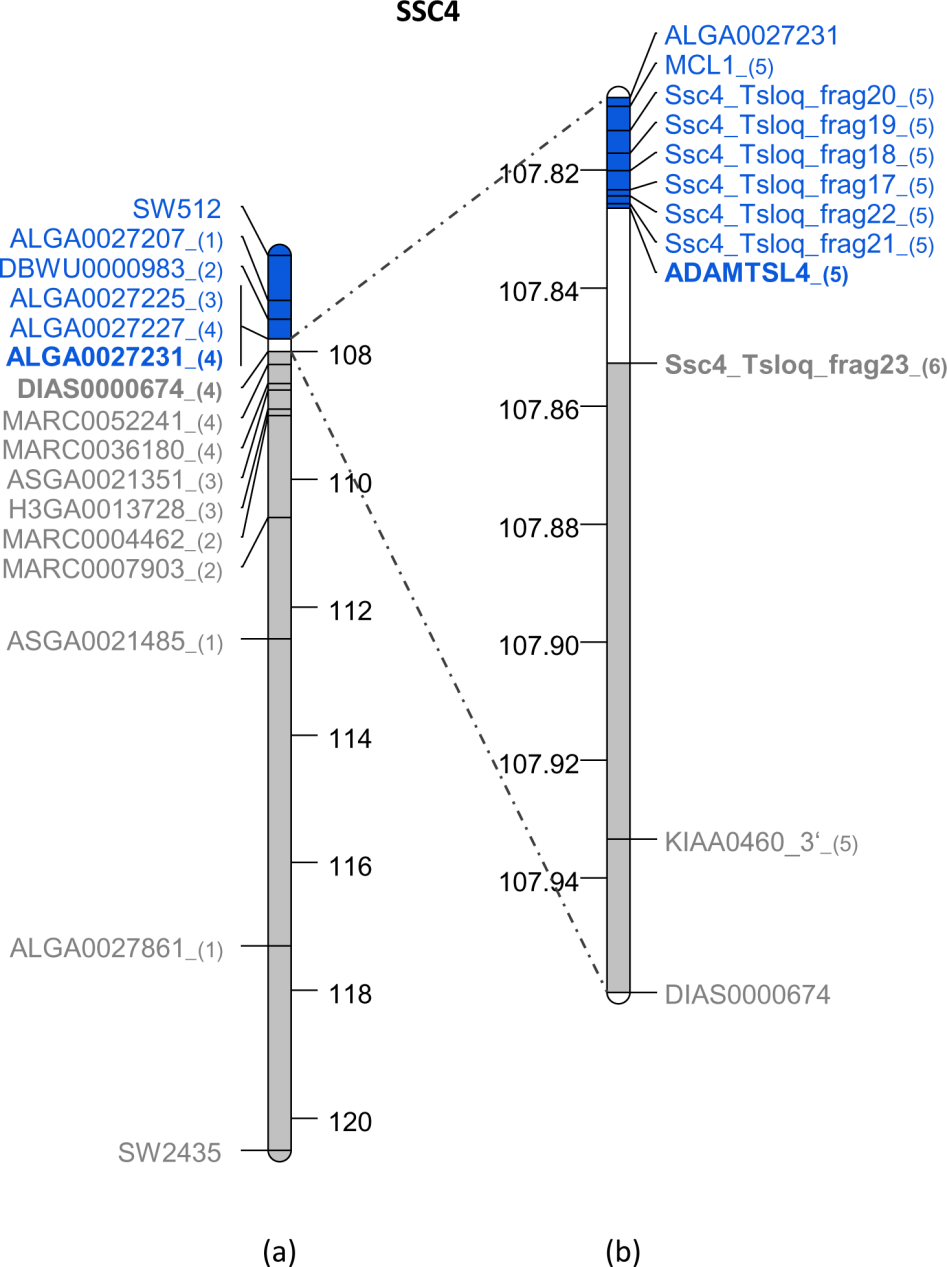
Map of the SSC4 region containing the breakpoint. All the markers (microsatellites, SNPs and STS) used in each round of PCR amplification are indicated on the map corresponding to the 106.5–120.5 Mb interval (a) or on a zoom of the 107.80–107.96 Mb interval (b). The successive batches of STS are indicated by a number in brackets following the STS names. Screening results obtained for each marker on Hb1.8 are represented by colors (blue: positive, grey: negative). The smallest interval on the SSC4 map is highlighted in white and the names of the two adjacent STS are indicated in bold.

For the two other microsatellites that were not mapped on the draft, the positions of the closest microsatellites on the genetic map were used as reference: SW251 (next to SW2527) was localized at 21.948 Mb on SSC3 and SW2435 (next to SW524) was localized at 120.509 Mb on SSC4 (Table 1). Using these data, intervals of less than 9.31 Mb on SSC3 and 14 Mb on SSC4 were thus defined. Because no additional microsatellite markers were available on the pig genetic map, the next step was performed using SNPs from the porcine 60K SNP bead-chip. Among the 60,000 SNPs on this chip, 249 were identified as potential STSs in the SSC3 interval and 398 in the SSC4 interval. A similar, iterative strategy was then used for both chromosomes: at each round, two to four STSs were selected to divide the current interval into three to five equal sub-intervals and PCR amplifications were performed using DNA of the Hb1.8 and Hb2.5 hybrid clones. PCR conditions are described in S1 Table (S1 Table). In each round, a smaller interval containing the breakpoint position was defined between two successive markers with opposite retention patterns, the first one giving an amplification product and the second one none using Hb1.8 DNA as template. In the next round, new STSs were then selected within the new interval defined. Using this step-by-step screening strategy, we were able to refine the localization of the breakpoints to between 23.803 and 23.876 Mb (interval size = 73.733 kb) on SSC3 (Fig 3A) and to between 107.808 and 107.959 Mb (interval size = 151.629 kb) on SSC4 (Fig 4A). No other SNPs were available within these intervals. New markers were defined based on the published porcine draft sequence (Sscrofa10.2) using a comparative mapping strategy.

### Comparative mapping and fine mapping of the breakpoint

In order to control the quality of the pig draft sequences in the two intervals of interest, the porcine sequences (pig Sscrofa10.2 assembly) were compared to the human draft genome sequence (human GRCh38/hg38 assembly). On SSC3, we observed a collinear organization between the 23.803–23.876 Mb interval on SSC3 and the 22.764–22.831 Mb interval on HSA16. Seven new STS markers were designed from the pig draft sequence and PCR were performed with Hb1.8 DNA as template (Fig 3B). The minimum interval was defined between the SSC3_Tsloq_frag29 and SSC3_Tsloq_frg30 loci, which mapped to the same BAC clone CH242-117J1 (accession number CU928801). Five additional STSs were defined from the BAC sequence and a short interval of 968 bp containing the breakpoint was defined on SSC3 between the SSC3_Tsloq_frag29 and SSC3_Tsloq_frag32 loci (Fig 3B).

On SSC4, the comparison of the Sscrofa10.2 assembly between positions 107.808 Mb and 107.989 Mb to the human genome sequence showed conserved synteny with the 150.417–150.555 Mb interval on HSA1 but in the opposite orientation. This alignment also revealed a missing sequence on the pig sequence draft between positions 107.854 and 107.904 Mb. To accurately define the position of the junction on SSC4, 12 primer pairs were selected, six in STSs that were defined from the available Sscrofa10.2 assembly (Ssc4_Tsloq_frag17, 18, 19, 20, 21 and 22), and six from porcine EST sequences corresponding to genes located in the orthologous human interval, including the *ECM1* gene located in the missing sequence (S1 Table). The localization of the breakpoint was refined to a region between the *ADAMTSL4* and *ECM1* genes that are separated by 52.924 kb on the human genome sequence. Finally, a STS marker (Ssc4_Tsloq_frag23) was selected on the pig BAC clone CH242-160A10 (accession number CU655899), which contains *ADAMTSL4* and was used to reduce the breakpoint interval on SSC4 to 20 kb (Fig 4B).

### Characterization of the breakpoint

By applying this chromosome walking approach, we identified the two most proximal positive loci obtained with the Hb1.8 clone, on both sides of the breakpoint, i.e. SSC3_Tsloq_frag29 on SSC3 and *ADAMTSL4* on SSC4. In order to bridge the breakpoint region, the four possible crossed-combinations of oligonucleotides (combining one primer for the SSC3_Tsloq_frag29 locus and one primer for *ADAMTSL4*) were tested on Hb1.8 DNA. One primer combination yielded an amplification product of approximately 1 kb. This product was purified, directly sequenced, and the sequences were compared to identify precisely the junction between the two chromosomal segments. The der(4) breakpoint was precisely mapped on SSC3 to position 48,007 Mb within the sequence of clone CH242-117J1 (GenBank accession number CU928801) and on SSC4, to position 27,558 Mb within the sequence of clone CH242-160A10 (GenBank accession number CU655899). To validate this result, a pair of primers was selected from the two BAC sequences on both sides of the breakpoint to amplify the junction der(3) using Hyb25.1 DNA as template. The resulting PCR product was sequenced and compared to the BAC DNA sequence. The breakpoint sequence obtained matched to the previously obtained alignment except for a small deleted sequence of 5 bp (TACAC) (S1 Fig). Using this information, we designed PCR primers on both sides of the breakpoint on SSC3 and 4 to amplify this region from the DNA of different animals (S1 Fig). Products of the expected size were obtained, which showed that the translocation could be genotyped from genomic DNA (S2A Fig).

### Translocation breakpoint mapping by next-generation sequencing

We used data from a parallel study on the detection of structural variations (SVs) in pigs to confirm the translocation breakpoint. In that study, samples from different animals were pooled to detect breed-specific SV. A heterozygous carrier of t(3;4)(p1.3;q1.5) was included in a pool of five Large White animals as a test case for the detection of SVs. Three SV-finding computational tools were tested on the generated sequencing data (see Materials and Methods). The translocation was identified with the three tested software but among a very large number of putative translocations (more than 500 for SVDetect and delly). The translocation was readily detected only if the analysis was limited to SSC3 and 4, with 34 supporting mate-pairs, among a few (4 or 5) translocation candidates (SVDetect and delly, respectively). GASVPro identified the translocation as the only reciprocal translocation when the analysis was limited to SSC3 and 4 but failed to find it when looking for translocations genome-wide. The differential case/control design, used to identify common and sample-specific SVs between the LW sample with the heterozygous carrier and a reference pool of Duroc samples, reduced the number of SV candidates to less than 100 putative translocations genome-wide and to a single translocation when limiting those candidates to translocations between SSC3 and 4. The breakpoints on both chromosomes were characterized with a resolution of 1.5 kb (23,832,968 bp– 23,834,409 bp) on SSC3 and 200 bp (107,826,800 bp– 107,826,982 bp) on SSC4.

### Expression analysis of the *ADAMTSL4* gene

The two translocated sequences were annotated using the http://www.ensembl.org/Sus_scrofa/blastview website. On SSC3, an intergenic small interspersed nucleotide element (SINE) retrotransposon spans the breakpoint and on SSC4, the breakpoint lies in intron 15 of the *ADAMTSL4* gene, resulting in the loss of the four last exons of the gene (S3 Fig). In order to assess whether expression of the gene was affected, we performed RT-PCR using primers that target exons 15 and 17, on each side of the breakpoint. As control, an additional pair of primers was designed in exons 13 and 14 (S3 Fig). Since these two exons are present in the native and the truncated sequence of the gene, amplification products should be obtained regardless of the genotype of the tested animals. These amplifications were performed on cDNA extracted from heart and lung tissues obtained from one homozygous translocated, one heterozygous and one non-affected control animal. As expected, there was no amplification from the translocated copy of the gene with the couple of primers defined in exons 15 and 17 (S2B Fig), confirming that expression of *ADAMTSL4* is indeed lost in the t(3;4) homozygous carrier.

## Discussion

**Fig 5:**
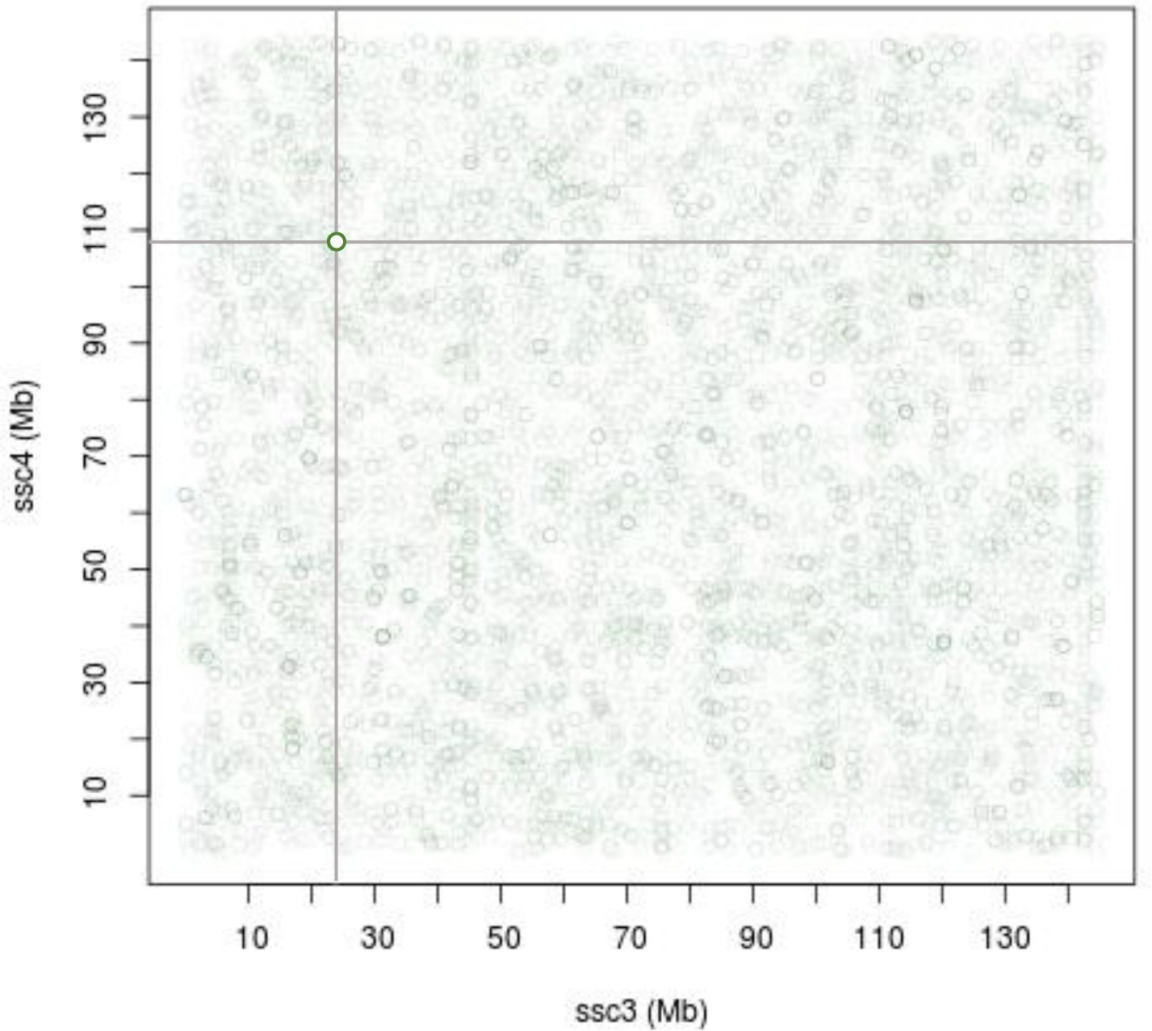
Distribution of discordant mate-pairs linking SSC3 to SSC4. Each circle represents a single mate-pair with one read mapped to SSC3 (x-axis) and the other read mapped to SSC4 (y-axis). In total, more than 12,000 mate-pairs link the two chromosomes, underlining the high background noise due to false-positive discordant mate-pairs. The dark green circle, where the two dashed lines cross, highlights a concentration of mate-pairs linking the two regions involved in the translocation.

One central goal of genome analyses is the comprehensive identification of all the genes together with their functions in the relevant biological processes. Studying chromosomal translocation breakpoints can provide insight into the function of genes when the translocation event disrupts their sequence. Here, we report the delineation and sequencing of a chromosomal breakpoint associated with a t(3;4)(p1.3;q1.5) reciprocal translocation that was detected in the Large-White pig line. We chose to characterize as finely as possible the breakpoints using somatic cell hybrids. While these are without doubt a powerful tool, constructing different panels for each translocation that occurs in pig populations is not feasible. Thus, we assessed the relevance of a high-throughput sequencing strategy using a pool of individuals including one translocation carrier. Our findings underline the fact that genome-wide screening for translocations, and more generally structural variants, using mate-pair sequencing on pools of individuals is not straightforward for various reasons. First, the status of the current pig genome reference assembly (SusScrofa 10.2) is certainly responsible for a large number of predicted structural variants that have no biological basis as attested by the significant reduction in the number of predicted SVs when using a differential case/control approach. A second reason arises from the fact that the genome that harbors the structural variant is pooled with “normal” genomes that hinder the detection of the translocation signal. Moreover, the signal associated with structural variants is known to be affected by high background noise due to false-positive discordant mate-pairs [28] (Fig 5). Thus, the detection of such translocation signals from pooled DNA samples requires dedicated bioinformatics approaches (currently under investigation). We would like to stress that the identification of structural variants—here a reciprocal translocation—using paired-end or mate-pair sequencing data is a feasible approach at the local level regardless of the size of the segments involved. The ability to detect such an event relies only on the existence of read pairs, here mate-pairs, which span the translocation breakpoints. This contrasts with the cytogenetic approach for which the ability to detect such events depends strongly on the size of the segments involved in the rearrangement. Here, starting from the initial characterization of the translocation, by combining somatic hybrid clone-based maps and sequencing, we obtained the precise sequences of the two implicated genomic regions, and defined accurately the breakpoints within the *ADAMTSL4* gene.

ADAMTS (a disintegrin and metalloproteinase with thrombospondin motifs) and ADAMTSL (a disintegrin and metalloproteinase with thrombospondin motifs-like) proteins are essential for the regulation of the development and maintenance of the extracellular matrix (ECM) as revealed by the clinical manifestations of Mendelian disorders resulting from mutations in each gene of these two families [29,30]. ADAMTS-Like 4 protein contains seven thrombospondin type 1 repeat (TSR1) domains, a cysteine-rich module, an ADAMTS-spacer 1 domain and a PLAC (Protease and LACunin) domain [31,32]. Six of the seven TSR1 domains are clustered towards the C end and are thought to have a role in anchoring ADAMTS-like proteins to the ECM [32]. A second truncated isoform of the protein comprising only three C-terminal TSR1 domains has also been described [31]. In humans, mutations in *ADAMTSL4* are clearly causative for ectopia lentis (EL) and congenital abnormalities of the iris [33–35]. In vitro, ADAMTSL4 has been characterized as a secreted glycoprotein that co-localizes with fibrillin-1 microfibrils and enhances fibrillin-1 microfibril deposition in the ECM of cultured fibroblasts [36]. Microfibrils constitute the principal component of the ocular zonule, which anchors the lens in its central position at the front of the eye and mediates accommodation [37]. Disruption of this cell-free rigging is the most current phenotype associated to EL. Among the different mutations identified to date, most of the variants appear to result from the truncation of the six C-terminal TSR1 domains which may prevent the protein from anchoring to the ECM [38]. In our study, the translocation point was located between exons 14 and 15, which should result in a truncated mRNA. After translation, the hypothetical protein would be similar to the natural isoform 2 of ADAMTSL4, with removal of the three last TSR1 and PLAC domains only. No visible pathogenetic or congenital defects were observed between affected (homozygous and heterozygous carriers) and non-affected animals during the selection of the line. However, it is important to note that visual acuity is not measured in this species and none of the defects (cataract, myopia, or retinal detachment) associated with EL in humans were tested in these animals. Even if the truncated protein produced is similar to the natural isoform 2 protein, the absence of isoform 1 in homozygous carriers may be of functional incidence. Thus, it is necessary to perform additional ophthalmologic testing. It is also important to note that many diseases that affect vision appear at a late stage in humans. In animal production, pigs are slaughtered at an early physiological age and this also might explain why no differences were observed among animals of different genotypes. To determine whether t(3;4) (p1.3;q1.5) translocation pig carriers could be used as a model for the study of some EL human diseases, it would be necessary to follow the animals over a longer period of time.

Among the animals of the line analyzed here, sows and boars that were pre-selected as future founders for the next generation were systematically analyzed by G-band karyotyping. Among the 110 candidates of the current generation, 27 heterozygous or two homozygous individuals were detected. Based on the precise identification of the translocation from this study, we developed a simple PCR diagnostic test to determine the genotype of the animals for this translocation. PCR amplifications were performed for all sires and dams from all generations since the creation of the line. Among the 636 reproducers, 42 heterozygous and one homozygous individuals were identified. The founder animal, which is responsible for the dissemination of the translocation, is one of the G0 founder boars. At each generation, six to 13 carrier individuals were selected, which gradually led to an increase in the frequency of the translocation in the line and to the occurrence of the first homozygous animal at generation 4.The effect of the translocation on litter size was confirmed *a posteriori*. The prolificacy of sires, which are heterozygous for the translocation, was 26% (P<2.10^−16^) lower than the performances of non-affected sires. Animals carrying the translocation in a homozygous state should not be impacted regarding their prolificacy or the segregation of chromosomes at meiosis since they produce only balanced gametes. Nevertheless, the number of homozygous animals is too small to test this hypothesis. With the current available data, it is not possible to determine whether the change in frequency is only due to genetic drift. An alternative hypothesis could be that the translocation itself or a quantitative locus localized near the translocation on SSC3 or 4 could have a favorable effect on a quantitative trait that is taken into account in the selection of this line. To test this hypothesis, it would be interesting to perform an association study between the genotypes obtained by diagnostic testing and the phenotypic traits recorded for the animals of the linen. A significant association would indicate that over the generations, the frequency of this translocation increased as a result of selection.

In conclusion, here we describe the fine characterization, at the base pair level, of a reciprocal translocation identified in a pig line. This work was carried out using a positional cloning (and chromosome walking) strategy in somatic cell hybrids generated from one animal carrying the translocation. This approach was successful but it is too time-consuming to be generalized for mapping other reciprocal translocations in pigs. The parallel mated-pair approach is the most rapid and powerful tool for characterizing and mapping translocations at the nucleotide level. In the future, systematic characterization of translocation points associated with the phenotypic analysis of carrier animals could contribute, as in humans, to a better understanding of the function of the genes affected by chromosomal rearrangements.

## Supporting information

S1 Table List of primer pairs used for the amplification of the different STS on Hb1.8 DNA. Positions of the primers on the reference sequence and PCR conditions are reported.
(PDF)

S1 Fig Alignment of the sequences generated from the translocated chromosomes with SSC3 and SSC4 reference sequences. Alignment of the der(3) translocated sequence obtained from Hb25.1 (in blue) and the der(4) translocated sequence obtained from Hb1.8 (in yellow) with the SSC3 genomic reference sequences (a) and the SSC4 genomic reference sequence (b). On SSC3 alignment, the 5-bp missing motif is colored in yellow. Positions of the four primers selected to perform the validation and the genotyping of the translocation are indicated in boxes.
(PDF)

S2 Fig PCR validations. (a) PCR validation on genomic DNA, according to the genotype of the individuals for the translocation. Primers were selected on each side of the translocation point to generate PCR products of different sizes depending on the amplified copy (Translocated vs Not-translocated).
(b) Amplification results obtained with two pairs of primers selected in exons 13–14 and exons 15–17 on lung and heart cDNA samples from three animals with different genotypes for the translocation. No amplification was observed with the Ex15_17 pair, which overlaps the translocation point, in animals that are homozygous for the translocation.
(PDF)

S3 Fig Schema of the *ADAMTSL4* gene. Schematic representation of the intron-exon and protein structures of the *ADAMTSL4* gene, based on data reported in [28] The different protein domains are shown, as well as the position of the translocation point (red arrow). The red dotted line indicates the portion of the protein removed by the reciprocal translocation (part of the fifth TSP1 domain, TSP1 domains 5 and 6 and the PLAC domain). The positions of the primers selected for validation on cDNA samples are reported on the genomic representation of *ADAMTSL4* (black arrows).
(PDF)
