# Supplementary material for: Identification of a t(3;4)(p1.3;q1.5) translocation breakpoint in pigs using somatic cell hybrid mapping and high-resolution mate-pair sequencing
Source: PLoS One. 2017 Nov 9;12(11):e0187617. doi: 10.1371/journal.pone.0187617 (PMC5679599; doi:10.1371/journal.pone.0187617)
Supplement: S1 Table — Positions of the primers on the reference sequence and PCR conditions are reported. (PDF) [file pone.0187617.s001.pdf]

STS				Primers								PCR conditions		
Name	Chromosome	Position (draft V10.2)	Acc Num (Probe database)	Acc Number (chromosome draft sequence)	Strand	Forward	Position from…	…to	Reverse	Position from…	…to	T°m	[ ] MgCl2	Cycles number
SW251	SSC3	21.948	Pr012488122			CCCAATATTCATAGCAGCATTG			TGAGTAATAGCCCATTTTGCG			56	1,5	36
H3GA0009045	SSC3	23.183		CM000814.4	+	TTAAACCTGTATGCCATGCTT	23 183 477	23 183 497	TGCTCACACACATGCAGAG	23 183 610	23183628	55	1,5	32
ALGA0107618	SSC3	23.388		CM000814.4	-	TCATCTTTAGACTTGGAATTGG	23 387 742	23 387 763	GAGTGTACTTCTTATTTCTGGCTTC	23 387 688	23 387 712	55	1,5	32
MARC0003844	SSC3	23.802		CM000814.4	+	AGTCCAGTGGGTTTCCAGAG	23 802 689	23 802 708	GATTCCCTGGGCGATTTAG	23 802 819	23 802 837	55	1,5	32
SSC3_Tsloq_frag1	SSC3	23.805		CM000814.4	+	TCTGCTTCCAATCACACAGC	23 805 383	23 805 402	TCCCAGTTTCCTCCTCAAAG	23 805 525	23 805 544	55	1,5	32
SSC3_Tsloq_frag28	SSC3	23.816		CM000814.4	-	GGCTCAGACATGAACTTTGC	23 816 294	23 816 313	CGAAGACGGTGATTCTTACG	23 816 225	23 816 244	55	1,5	32
SSC3_Tsloq_frag25	SSC3	23.832		CM000814.4	-	CCAGCTACCCAGCCTCATAG	23 832 176	23 832 195	GCCTCGTAGACCGAATACTCC	23 831 979	23 831 999	55	1,5	32
SSC3_Tsloq_frag29	SSC3	23.834		CM000814.4	+	TGATTCCCTGGTGATCCAAC	23 833 752	23 833 771	AATGGAGAAGAGCCCAAAGG	23 833 874	23 833 893	55	1,5	32
SSC3_Tsloq_frag32	SSC3	23.834		CM000814.4	+	AGGCACTGAAGCCACACAG	23 834 572	23 834 590	TGGGCAAAAGACATGAAAAG	23 834 695	23 834 714	58	1,5	32
SSC3_Tsloq_frag33	SSC3	23.835		CM000814.4	+	CCACAGAATCCTAGCTCGTG	23 835 320	23 835 339	TGATGACTTATTATACAGCCGTTAG	23 835 383	23 835 407	58	1,5	32
SSC3_Tsloq_frag34	SSC3	23.836		CM000814.4	+	GCCCTGGACCTCTGTACTTG	23 836 268	23 836 287	GGGAGAGAGACTGGACCAAAG	23 836 338	23 836 358	58	1,5	32
SSC3_Tsloq_frag35	SSC3	23.837		CM000814.4	+	CCCTTGACCACACTTTGGAG	23 837 019	23 837 038	TTCGGGTTCAAATCTCTTGG	23 837 143	23 837 162	55	1,5	32
SSC3_Tsloq_frag36	SSC3	23.838		CM000814.4	+	ATTGACACTGGGCAAGAAGG	23 837 799	23 837 818	TTAACCACTGCACCACAACG	23 837 867	23 837 886	55	1,5	32
SSC3_Tsloq_frag30	SSC3	23.839		CM000814.4	+	TTCCTGCCTCTCTCTGAACC	23 838 812	23 838 831	GAGTCAACCGTGGGATGTG	23 838 884	23 838 902	55	1,5	32
SSC3_Tsloq_frag5	SSC3	23.847		CM000814.4	+	CATTCATCACGGGTCTGTTG	23 847 151	23 847 170	ACCAAACCACCCCAATCTAC	23 847 330	23 847 349	55	1,5	32
SSC3_Tsloq_frag6	SSC3	23.874		CM000814.4	+	ACTCGTATGGGTGAGGAAGG	23 874 357	23 874 376	ACAACCTGCACAACCTGATG	23 874 559	23 874 578	55	1,5	32
SIRI0001150	SSC3	23.876		CM000814.4	-	ACCATCCTGCTGCATTTCTC	23 876 677	23 876 696	AGCTGGGGAAGAGGCTTTAG	23 876 514	23 876 533	58	1,5	34
ALGA0123020	SSC3	24.043		CM000814.4	+	ACCCAGAGACCAATCAGCAC	24 042 514	24 042 533	TTTATTGCACGTTGCCTTTG	24 042 649	24 042 668	58	1,5	34
ASGA0097395	SSC3	24.214		CM000814.4	+	TCCCATCTCTTCTCCTGCAC	24 214 399	24 214 418	GGTTTGTGCCCTATTGGATG	24 214 511	24 214 530	58	1,5	34
ASGA0094620	SSC3	24.288		CM000814.4	-	TTTGATGAACCAAGCCAGGTAA	24 288 121	24 288 142	TAAAACACATCTAGTACCGCAGACA	24 288 044	24 288 068	58	1,5	34
ALGA0121834	SSC3	24.364		CM000814.4	+	ACTTATGCTGAGCCACGATG	24 364 053	24 364 072	TCCAGGGATTTCTCAGTTGG	24 364 182	24 364 201	58	1,5	34
ALGA0018079	SSC3	25.957		CM000814.4	-	GACCTGATACCGTAGTTGGAGAC	25 957 486	25 957 508	AATCGCAGTTGGAGAGGTTC	25 957 314	25 957 333	58	1,5	32
M1GA0004189	SSC3	26.724		CM000814.4	+	GATGCTCCCCTGTTTGGTC	26 724 365	26 724 383	AATGGGTGCGTGCTTTTC	26 724 527	26 724 544	58	1,5	32
SW487	SSC3	31.261	Pr012488476			TGAGCACCTCTGCTTGAGTC			ACACCTCTAAAATGGCAGTGTG			55	1,5	36
SW512	SSC4	106.511	Pr012488478			TATAGTGCAGTTATATCTCAATACAAATGG			TCTGACATTAATACAACCACCCC			50	1,5	40
ALGA0027207	SSC4	107.248		CM000815.4	+	AAGGGCAGGGAAGGTCAG	107 248 294	107 248 311	TGTTTGCTTCTCCCAAGTCC	107 248 454	107 248 473	58	1,5	32
DBWU0000983	SSC4	107.485		CM000815.4	-	AACTGAACTCAAACCCTGAAACA	107 485 143	107 485 165	CTGTCTCCACACATCTCCCTTA	107 485 065	107 485 086	58	1,5	35
ALGA0027225	SSC4	107.759		CM000815.4	+	AGTCTCTGGGTTTGGGGTCT	107 759 615	107 759 634	AGCAAATGCCAGTTAATTTGTAAG	107 759 739	107 759 762	58	1,5	35
ALGA0027227	SSC4	107.781		CM000815.4	+	GGGAAAGGTGGTTAGGGAGA	107 780 781	107 780 800	CACCTGGTTCAAGATCAGCA	107 780 876	107 780 895	55	1,5	32
ALGA0027231	SSC4	107.808		CM000815.4	+	AAAGTGAAACCAGCAATGCAC	107 807 849	107 807 869	TTCGGCTAGGAGAGCTAGGG	107 807 994	107 808 013	58	1,5	34
MCL1	SSC4	107.809		CM000815.4	+	TTTTCTGCCAAGGTGGTG	107 809 227	107 809 244	TCTTTTCCCGTAGCCAAGAG	107 809 366	107 809 385	50	1,5	40
Ssc4_Tsloq_frag20	SSC4	107.813		CM000815.4	-	GGTCACATTCTGAGGCTGAAG	107 813 286	107 813 306	CCTGTCTTTGGGGAGATAAAAG	107 813 083	107 813 104	58	1,5	30
Ssc4_Tsloq_frag19	SSC4	107.817		CM000815.4	-	GGATTGGACCCACAACCTC	107 817 065	107 817 083	CACAACCTGGAGTTCCCATC	107 817 010	107 817 029	65	1,5	32
Ssc4_Tsloq_frag18	SSC4	107.820		CM000815.4	-	GGTAAAGCACTTGCCATGTATTC	107 820 009	107 820 031	TCTCAGGCCCTAATTTTCTCC	107 819 934	107 819 954	58	1,5	30
Ssc4_Tsloq_frag17	SSC4	107.823		CM000815.4	-	CTGAGCCTGAGAACCAATCAC	107 823 221	107 823 241	GAGAAGGGAGGTCCTGAAGC	107 823 162	107 823 181	58	1,5	30
Ssc4_Tsloq_frag22	SSC4	107.824		CM000815.4	-	TTTCCTGTTCCCTTCCTTTG	107 824 357	107 824 376	CTCCACCCTCGACTCCAC	107 824 173	107 824 190	58	1,5	32
Ssc4_Tsloq_frag21	SSC4	107.826		CM000815.4	-	GGCTCTGCGTCTATCCCTAC	107 825 637	107 825 656	GGTACAGAGTGGGCTGGTTG	107 825 494	107 825 513	58	1,5	32
ADAMTSL4	SSC4	107.826		CM000815.4	-	GCACACAGCGTAGAGACATC	107 826 393	107 826 412	CAGGGTGTAGAAAACCATCG	107 826 262	107 826 281	66	1,5	34
Ssc4_Tsloq_frag23	SSC4	107.853		CM000815.4	-	CAGATGGAGGCTGAGTTGG	107 852 278	107 852 296	CCACTTGCTGATTGCTGAGA	107 852 166	107 852 185	58	1,5	34
ECM1	SSC4	?		XM_021089911.1	+	TGAGCCCAAGGAAGAGTGAG	1 357	1 376	GGAGGGTGTGTTTGTGTGTG	1 510	1 491	60	1,5	32
KIAA0460_3'	SSC4	107.933		CM000815.4	-	ATAGAGGACATGGGCGTGAG	107 933 184	107 933 203	CGTTCCTTGTACGGTGCTG	107 933 041	107 933 059	53	1,5	35
DIAS0000674	SSC4	107.959		CM000815.4	-	CCCTGGTCTGTGTTCAGTCTC	107 959 520	107 959 540	GCATTTGAGAGTTTCTGTGCTG	107 959 404	107 959 425	65	1,5	34
KIAA0460_Ex3	SSC4	107.983		CM000815.4	-	CTGCCTATCCTCACCGTTTG	107 982 758	107 982 777	AGATCCCAAAGGGCAACAAG	107 982 617	107 982 636	55	1,5	32
PRPF3	SSC4	108.033		CM000815.4	+	GGGAAGGAAGCAAGGTTTTC	108 033 046	108 033 061	CCGACTGAGACTACTGCAAGC	108 033 184	108033204	50	1,5	40
MARC0052241	SSC4	108.209		CM000815.4	+	TGAGGGATTTGCAGACACAC	108 208 848	108 208 867	CAGTGCAGCAGGGAGAGATAC	108 208 977	108 208 997	58	1,5	34
MARC0036180	SSC4	108.479		CM000815.4	+	CAACCTTTCCCTCGTTACTCC	108 478 912	108 478 932	AAAGTCCACCATCCCTCTCC	108 479 039	108 479 058	58	1,5	34
ASGA0021351	SSC4	108.588		CM000815.4	-	TCTCAGACAAAGAAGAGAAGCTCA	108 588 221	108 588 244	CTCATCCTGCCTCCAACTCT	108 588 118	108 588 137	58	1,5	34
H3GA0013728	SSC4	108.900		CM000815.4	+	TCTCAGCAAGAGAAGCACGA	108 900 212	108 900 231	CACTGAAGAAAGGAACGTGGA	108 900 317	108 900 337	58	1,5	32
MARC0004462	SSC4	109.015		CM000815.4	-	CCTTCGCTAGTGTCCATGAG	109 015 731	109 015 750	TAGCAGGAAGGAGAGGGTGA	109 015 594	109 015 613	58	1,5	35
MARC0007903	SSC4	110.606		CM000815.4	+	TGCACACACTAAATGGTCATAGGT	110 606 229	110 606 249	CCAGATAGACGGCTGATAGATG	110 606 375	110 606 394	58	1,5	32
ASGA0021485	SSC4	112.476		CM000815.4	-	CCGTCTCTAGCGTCTCCATT	112 476 413	112 476 432	CACCTGCTCTGGTTGGAAGT	112 476 267	112 476 286	58	1,5	32
ALGA0027861	SSC4	117.316		CM000815.4	+	AGAGGAGAACCCCTTGCAGT	117 315 687	117 315 706	CAAAAGTAACCAACCCAGCAG	117 315 834	117 315 854	58	1,5	32
SW2435	SSC4	120.509	Pr009672132			CAAAGCAGATGCACAGTTTAGG			CAGAGGGTTGTTGGTGGG			55	1,5	40

S1 Table
