# Supplementary material for: Identification of a t(3;4)(p1.3;q1.5) translocation breakpoint in pigs using somatic cell hybrid mapping and high-resolution mate-pair sequencing
Source: PLoS One. 2017 Nov 9;12(11):e0187617. doi: 10.1371/journal.pone.0187617 (PMC5679599; doi:10.1371/journal.pone.0187617)
Supplement: S1 Fig — Alignment of the der(3) translocated sequence obtained from Hb25.1 (in blue) and the der(4) translocated sequence obtained from Hb1.8 (in yellow) with the SSC3 genomic reference sequences (a) and the SSC4 genomic reference sequence (b). On SSC3 alignment, the 5-bp missing motif is colored in yellow. Positions of the four primers selected to perform the validation and the genotyping of the translocation are indicated in boxes. (PDF) [file pone.0187617.s002.pdf]

(a)

```
Hb1.8                                                                                                                                          C
                                                                                                                                               |
SSC3    CTTTTGTATGGTTGTGTTTTTTTTTCTTTGTTGTTGTTTTTTTTTTTTCTGGTCTTTTTGCCTTTTCTAGGGCCACTCGCCTTGGCATATGGAGGTTCCCAGGCTAGGGGTCCAGTCGGAGCCATAGC

Hb2.5                                                          cacatccaccacaccctctcagagcctctccaactcggttaaataaacttttattgagtaagcacctgctgtatgctagat
```

Trsl_SSC3.Up >

```
Hb1.8   TGCCAGCCTACAACCACAGCCACAGCAACTCAGGATCCGAGCCGCGTCTGCGACCtgccggcaaagtctccctgtatctggctggtacccctgggggcattccctatccccggtccagaatgtgtct
        |||||||||||||||||||||||||||||||||||||||||||||||||||||||
SSC3    TGCCAGCCTACAACCACAGCCACAGCAACTCAGGATCCGAGCCGCGTCTGCGACCTACACCACAGCTCACTGCAACGCCGGATCCTTAACCCACGGAGCAAGGCCAGGGATCGAACCCGAAACCTTA
                                                                    ||:||||||||||||||||||||||||||||||||||||||||||||||||||||||||||||||||
Hb2.5   gctgctgatgccgggaacttggagctaagtgaggaaaacttacattaagacccttgccccCATAGCTCACTGCAACGCCGGATCCTTAACCCACGGAGCAAGGCCAGGGATCGAACCCGAAACCTTA


Hb1.8   gtcctcagccccctcctcttcccctacctgtcacagccctctctgcttcccaacagtgctcagccgagtgtgggacgggaatccagcgacgctctgtggtctgccttgggagt

SSC3    TGGTTCCTAGTCCTTAACCACGGAGGCATGAAGGGAACTCCTGGATGTAAGTTTTCAT-TTCCTTGGGAACAATGGCCAAGAGCTCAGTTGCCAGCTCACATGGTAATTGCACATTTCGTATGTAAG
        ||||||||||||||||||||||||||||||||||||||||||||||||||||||||||:|||||||||||||
Hb2.5   TGGTTCCTAGTCCTTAACCACGGAGGCATGAAGGGAACTCCTGGATGTAAGTTTTCATCTTCCTTGGGAACA
```

<Trsl_SSC4.Dn

(b)

Trsl_SSC4.Up >

```
Hb1.8                                                                                                                                         C

SSC4    GGCCCCACACCCCCTCAGCCAAGTCCCCATCCCTGGTGCCCCACATCCACCACACCCTCTCAGAGCCTCTCCAACTCGGTTAAATAAACTTTTATTGAGCAAGCACCTGCTGTATGCTAGATGCTGC
                                                 ||||||||||||||||||||||||||||||||||||||||||||||||||||||||||||||||||||||||||||||||||||||
Hb2.5                                            CACATCCACCACACCCTCTCAGAGCCTCTCCAACTCGGTTAAATAAACTTTTATTGAGTAAGCACCTGCTGTATGCTAGATGCTGC


Hb1.8   tgccagcctacaaccacagccacagcaactcaggatccgagccgcgtctgcgaccTGCCGGCAAAGTCTCCCTGTATCTGGCTGGTACCCCTGGGGGCATTCCCTATCCCCGGTCCAGAATGTGTCT
                                                               ||||||||||||||||||||||||||||||||||||||||||||||||||||||||||||||||||||||||
SSC4    TGATGCCGGGAACTTGGAGCTAAGTGAGGAAAACTTACATTAAGACCCTTGCCCCTGCCGGCAAAGTCTCCCTGTATCTGGCTGGTACCCCTGGGGGCATTCCCTATCCCCGGTCCAGAATGTGTCT
        |||||||||||||||||||||||||||||||||||||||||||||||||||||||
Hb2.5   TGATGCCGGGAACTTGGAGCTAAGTGAGGAAAACTTACATTAAGACCCTTGCCCCcatagctcactgcaacgccggatccttaacccacggagcaaggccagggatcgaacccgaaaccttatggtt


Hb1.8   GTCCTCAGCCCCCTCCTCTTCCCCTACCTGTCACAGCCCTCTCTGCTTCCCAACAGTGCTCAGCCGAGTGTGGGACGGGAATCCAGCGACGCTCTGTGGTCTGCCTTGGGAGT
        |||||||||||||||||||||||||||||||||||||||||||||||||||||||||||||||||||||||||||||||||||||||||||||||||||||||||||||||||
SSC4    GTCCTCAGCCCCCTCCTCTTCCCCTACCTGTCACAGCCCTCTCTGCTTCCCAACAGTGCTCAGCCGAGTGTGGGACGGGAATCCAGCGACGCTCTGTGGTCTGCCTTGGGAGTGGGGAGGCCCATGG

Hb2.5   cctagtccttaaccacggaggcatgaagggaactcctggatgtaagttttcatcttccttggg
```

<Trsl_SSC3.Dn

S1 Fig
