# Supplementary figures and images for: Identification of a t(3;4)(p1.3;q1.5) translocation breakpoint in pigs using somatic cell hybrid mapping and high-resolution mate-pair sequencing
Source: PLoS One. 2017 Nov 9;12(11):e0187617. doi: 10.1371/journal.pone.0187617 (PMC5679599; doi:10.1371/journal.pone.0187617)

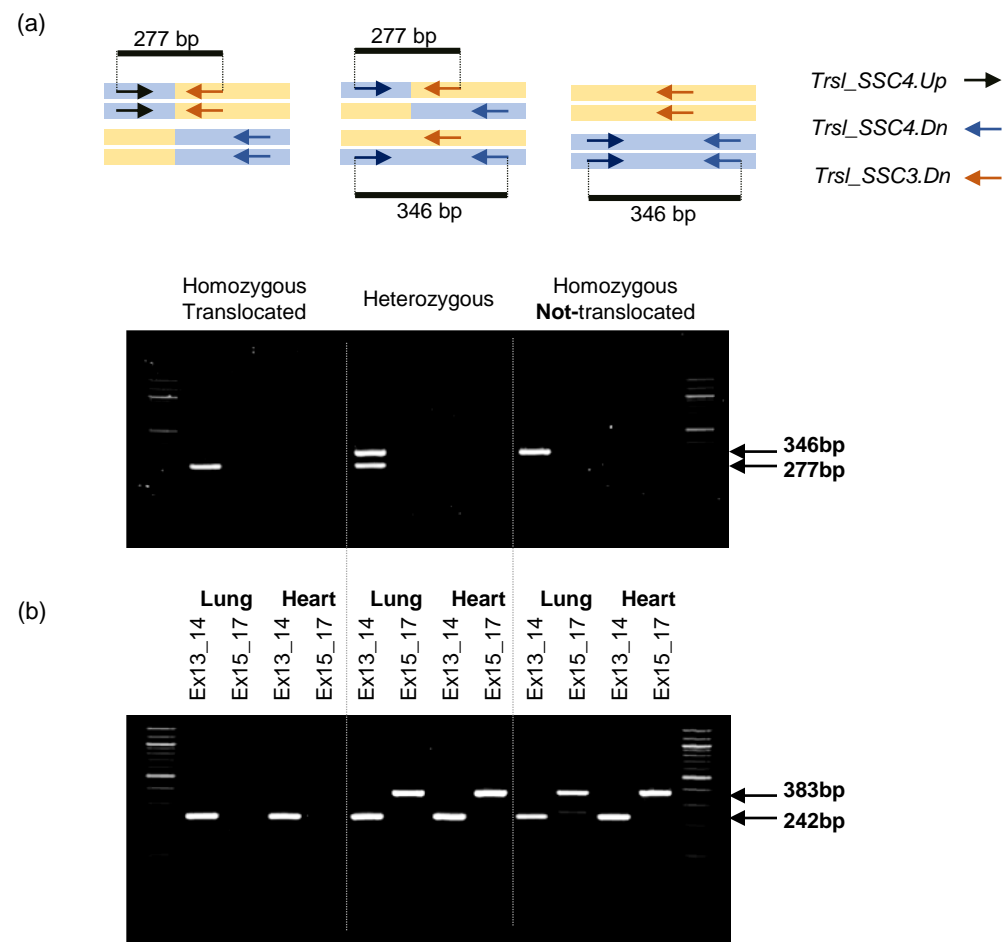

S2 Fig

Supplement: S2 Fig — (a) PCR validation on genomic DNA, according to the genotype of the individuals for the translocation. Primers were selected on each side of the translocation point to generate PCR products of different sizes depending on the amplified copy (Translocated vs Not-translocated). (b) Amplification results obtained with two pairs of primers selected in exons 13–14 and exons 15–17 on lung and heart cDNA samples from three animals with different genotypes for the translocation. No amplification was observed with the Ex15_17 pair, which overlaps the translocation point, in animals that are homozygous for the translocation. (PDF) [file pone.0187617.s003.pdf]

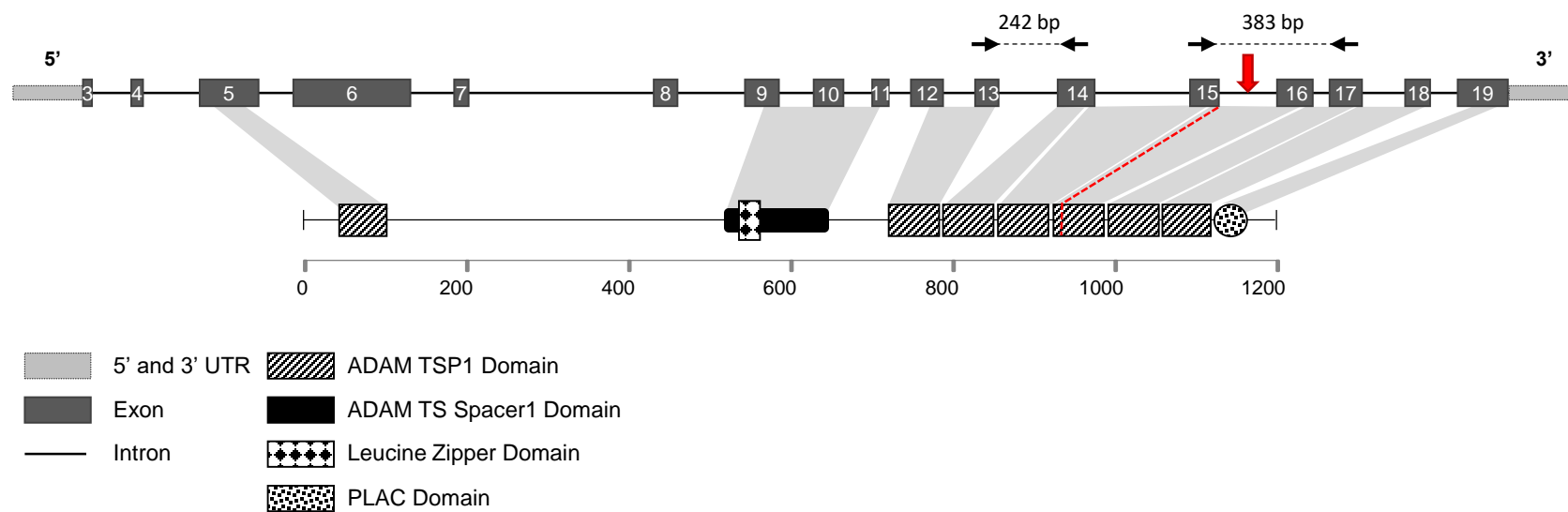

S3 Fig

Supplement: S3 Fig — Schematic representation of the intron-exon and protein structures of the ADAMTSL4 gene, based on data reported in [28] The different protein domains are shown, as well as the position of the translocation point (red arrow). The red dotted line indicates the portion of the protein removed by the reciprocal translocation (part of the fifth TSP1 domain, TSP1 domains 5 and 6 and the PLAC domain). The positions of the primers selected for validation on cDNA samples are reported on the genomic representation of ADAMTSL4 (black arrows). (PDF) [file pone.0187617.s004.pdf]
